# MiR-193b promotes autophagy and non-apoptotic cell death in oesophageal cancer cells

**DOI:** 10.1186/s12885-016-2123-6

**Published:** 2016-02-15

**Authors:** Michelle J. Nyhan, Tracey R. O’Donovan, Antonius W. M. Boersma, Erik A. C. Wiemer, Sharon L. McKenna

**Affiliations:** Cork Cancer Research Centre, 4th Floor Western Gateway Building, University College Cork, Cork, Ireland; Department of Medical Oncology, Erasmus MC Cancer Institute, Erasmus University Medical Center, Rotterdam, The Netherlands

**Keywords:** MiR-193b, Autophagy, Chemosensitivity, Stathmin 1, Oesophageal cancer, Non-apoptotic cell death

## Abstract

**Background:**

Successful treatment of oesophageal cancer is hampered by recurrent drug resistant disease. We have previously demonstrated the importance of apoptosis and autophagy for the recovery of oesophageal cancer cells following drug treatment. When apoptosis (with autophagy) is induced, these cells are chemosensitive and will not recover following chemotherapy treatment. In contrast, when cancer cells exhibit only autophagy and limited Type II cell death, they are chemoresistant and recover following drug withdrawal.

**Methods:**

MicroRNA (miRNA) expression profiling of an oesophageal cancer cell line panel was used to identify miRNAs that were important in the regulation of apoptosis and autophagy. The effects of miRNA overexpression on cell death mechanisms and recovery were assessed in the chemoresistant (autophagy inducing) KYSE450 oesophageal cancer cells.

**Results:**

MiR-193b was the most differentially expressed miRNA between the chemosensitive and chemoresistant cell lines with higher expression in chemosensitive apoptosis inducing cell lines. Colony formation assays showed that overexpression of miR-193b significantly impedes the ability of KYSE450 cells to recover following 5-fluorouracil (5-FU) treatment. The critical mRNA targets of miR-193b are unknown but target prediction and siRNA data analysis suggest that it may mediate some of its effects through stathmin 1 regulation. Apoptosis was not involved in the enhanced cytotoxicity. Overexpression of miR-193b in these cells induced autophagic flux and non-apoptotic cell death.

**Conclusion:**

These results highlight the importance of miR-193b in determining oesophageal cancer cell viability and demonstrate an enhancement of chemotoxicity that is independent of apoptosis induction.

## Background

Oesophageal cancer is a highly aggressive cancer with a poor 5-year survival rate (<20 %) [1]. Current treatment regimens fail to eradicate disseminated tumour cells which can re-establish as a drug resistant secondary cancer [2]. Our research group previously analysed a panel of oesophageal cancer cell lines and their response to treatment with standard chemotherapeutics (5-fluorouracil (5-FU) and cisplatin). Cells that were sensitive to these drugs were both apoptosis and autophagy competent and did not recover following withdrawal of the drug. In contrast, cells that were drug resistant exhibited only autophagy with limited Type II cell death and recovered following removal of the chemotherapeutics. Silencing of key autophagy regulators Beclin 1 and ATG7 verified the crucial role autophagy plays in this recovery process [3].

Autophagy is an evolutionary conserved catabolic process in which cells self-digest their own organelles, protein aggregates and other macromolecules to maintain cellular homeostasis. However, when a tumour has become established, cancer cells utilise autophagy as a mechanism to protect themselves from adverse stressful conditions including nutrient starvation, hypoxia and anticancer treatments [4]. Inducing excessive autophagy (Type II cell death) has also been identified as an important cell death pathway [5]. Autophagy with Type II cell death has been observed following the treatment of several different types of cancer with chemotherapeutics [6]. It is crucial that we have a clearer understanding of the signalling pathways required to enhance cell death associated autophagy and prevent autophagy that promotes survival of cancer cells.

The importance of microRNAs (miRNAs) in the regulation of diverse cellular processes implicated in tumourigenesis including proliferation, migration, angiogenesis, metastasis, apoptosis and autophagy has only begun to emerge. MiRNAs are small, non-coding RNA molecules of 22–24 nucleotides in length. They act as powerful negative regulators of gene expression (acting at the posttranscriptional and translational levels) and are believed to regulate at least 30 % of human genes. A single miRNA may target several mRNAs at once and consequently can act on complex regulatory networks. Depending on the cellular context and the availability of direct mRNA targets, the same miRNA may have quite diverse functions. MiRNAs have now emerged as potential targets to be exploited for anticancer drug development [7–10]. MiRNAs have been identified to have crucial roles in various stages of autophagy including induction (e.g. miR-101), vesicle nucleation (e.g. miR-30a), elongation and completion (e.g. miR-181a), docking and fusion (e.g. miR-34a) and degradation and recycling (e.g. miR-17/20/93/106 complex) [11–14]. Several miRNAs have also been implicated in the development of drug resistance [15]. However, as this remains an emerging field, there are likely to be numerous miRNAs, which have yet to be identified, that regulate cell survival/death mechanisms and thereby impact on chemosensitivity.

In this study, we present evidence that miR-193b has an important role in determining the chemosensitivity of oesophageal cancer cells. Overexpression of miR-193b significantly reduces the ability of cancer cells to recover following treatment with 5-FU. MiR-193b may mediate its effects, at least in part, through decreasing the expression of stathmin 1. Stathmin 1 is a ubiquitous cytosolic phosphoprotein that regulates microtubule dynamics. Its expression levels have previously been reported to influence the responsiveness of cancer cells to chemotherapeutics [16–18]. We found that increased expression of miR-193b does not activate apoptosis but elevates autophagy and non-apoptotic cell death.

## Methods

### Cell culture/reagents

Human oesophageal cancer cell lines OE19, OE21 and OE33 were obtained from the European Collection of Cell Cultures (96071721, 96062201 and 96070808). KYSE450 cells were from DSMZ (Deutsche Sammlung von Mikroorganismen und Zellkulturen GmbH, ACC-387). OE21, OE19 and OE33 cells were grown in RPMI 1640 medium (Sigma, R8758). KYSE450 cells were maintained in 50: 50 RPMI: F12 HAMS medium (Sigma, N6658), all supplemented with 10 % foetal calf serum (Sigma, F7524) and 1 % Penicillin/Streptomycin (Gibco-BRL, 15070-063) and grown at 37 °C, 5 % CO_2_. Chloroquine (C6628) and 5-FU (F6627) were purchased from Sigma.

### Microarray and data analysis

MiRNA expression profiling and data analysis was performed as previously described [19, 20]. The probe set contained 1344 probes capable of detecting 725 human miRNAs in addition to mouse, rat and viral probes. These experiments were carried out in triplicate. The microarray expression data have been deposited to the Gene Expression Omnibus (GEO) data repository (accession number GSE77132).

### Real-time PCR

RNA was extracted from all cell lines, cDNA synthesised and PCR performed as per manufacturer’s instructions (Qiagen, 217004, 218161 and 218073) using the Lightcycler system (Roche). MiR-193b (MS00031549) and RNU6B (used for normalisation; MS00033740) primers were designed and synthesised by Qiagen. Results represent at least three independent experiments. Expression levels were quantified using the ‘Delta-Delta’ formula [21].

### Transfections

MiR-193b mimics (C-300764-05-0010), scrambled miRNAs (negative control; CN-001000-01-05), stathmin 1 siRNA (L-005152-00-0005) and scrambled siRNAs (negative control; D-001810-01-20) were designed and synthesised by Dharmacon. Cells were transiently transfected using Lipofectamine 2000 (Invitrogen, 11668-027) according to the protocol provided with the reagent. SiGlo green transfection indicator (Dharmacon, D-001630-01-05) was used to assess transfection efficiency (which was always greater than 70 %).

### Western blotting

Protein extracts were lysed in modified RIPA buffer (50 mM Tris–HCl pH 7.4, 150 mM NaCl, 0.25 % sodium deoxycholate, 1 % Igepal, 1 mM EDTA, 1× PefaBloc, 1× Protease Inhibitor Cocktail, 1 mM Na_3_VO_4_, 1 mM NaF). Protein samples were separated on pre-cast NuPAGE 4–12 % Bis-Tris gels (Invitrogen, NP0322) and electrophoretically transferred onto PVDF membranes (Invitrogen, IB401001). Primary antibodies were: anti-stathmin 1 (Abcam, ab52630), anti-LC3 (MBL, PD014) and anti-actin (Sigma, A5441). Proteins were visualised using relevant IR-Dye secondary antibodies and quantified using the Odyssey Infrared imaging system (Li-Cor).

### Colony formation assay

Following treatment, all adherent cells were trypsinised and counted. Fifteen hundred viable cells were reseeded in fresh media (without drug) into a well of a six well plate (in triplicate) and allowed to grow for 10–14 days. Colonies were then fixed with 96 % ethanol and stained with ProDiff Solution C (Braidwood Laboratories, 22009). Plates were scanned using the Odyssey Infrared Imaging system (Li-Cor) and colonies quantified. Results are presented as integrated intensity ± standard error of the mean (SEM) from at least three independent experiments. When colony numbers were very low, colonies were manually counted.

### Flow cytometry assay

#### Autophagy

Cyto-ID Autophagy Detection Kit (Enzo Life Sciences, ENZ-51031) was used to stain live cells according to manufacturer’s instructions. This assay is dependent on a 488 nm-excitable green fluorescent detection reagent which specifically fluoresces in autophagic vesicles. An increase in the 488-2A channel represents an increase in the number of autophagic vesicles staining green.

#### Caspase 3 assay

Cells were fixed in 4 % paraformaldehyde and washed in permeabilisation buffer (0.1 % Triton–X-100, 0.1 % sodium azide, 10 mM HEPES, 4 % FCS, 150 mM NaCl). Subsequently, cells were incubated with anti-active caspase-3 antibody (BD BioSciences, 559565) and then with an anti-rabbit FITC conjugated secondary antibody.

BD-LSRII flow cytometer (BD BioSciences) was used to detect fluorescence. Data analysis and histogram overlays were done using the FlowJo software.

### Morphological examination of cell death

Following cytospinning onto glass slides, cells were stained with Pro-Diff (Braidwood Laboratories, 22007, 22008 and 22009). Morphology images were captured using a DP70 digital microscope camera and Olympus DP-Soft823 version 3.2 software. All images are representative of at least three independent experiments. Apoptosis is identified by shrinking of the cell, chromatin condensation and DNA fragmentation into ‘apoptotic bodies’ within an intact plasma membrane. Type II cell death was characterised by loss of cytoplasmic material, an increase in the number of cytoplasmic vesicles, pyknosis (condensed regions) of the nuclear material and an intact nuclear membrane [22].

### HMGB1 ELISA

Following treatment, supernatant was removed from cells and subsequently diluted 1:1 with dilution buffer. Measurement of High Mobility Group Box 1 (HMGB1) concentration was determined using the HMGB1 ELISA kit (Chondrex, 6010) according to manufacturer’s instructions.

### Statistical analysis

All statistical analysis was performed using GraphPad Prism version 5. Comparisons between groups were assessed using Student’s *t* test.

## Results

### MiR-193b is differentially expressed between chemosensitive and chemoresistant oesophageal cancer cells

We undertook miRNA expression profiling of a panel of oesophageal cancer cell lines which differ in their response to treatment with chemotherapy drugs. Two of these cell lines (OE21 & OE33 – Group A) induce apoptosis and autophagy and are relatively chemosensitive and two cell lines (KYSE450 & OE19 – Group B) respond by inducing autophagy with limited Type II cell death and have the ability to recover following removal of cytotoxic drugs [3]. The miRNA expression profile of Group A versus Group B was analysed on a microarray platform which consisted of 1344 LNA capture probes, of which 725 hybridise to annotated human miRNAs. In this analysis, 440 human miRNAs were expressed above background level. This screen allowed us to identify miRNAs which may be have a crucial role in the regulation of these diverse processes.

**Fig. 1:**
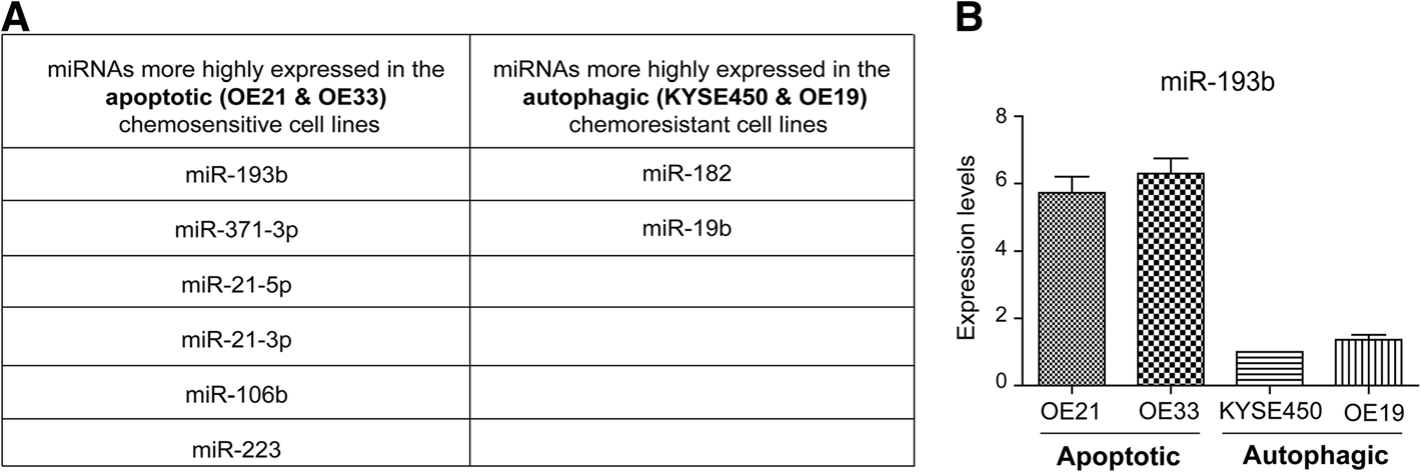
miRNAs differentially expressed between the chemosensitive and chemoresistant oesophageal cancer cell lines. **a** The table lists the miRNAs that are differentially expressed (≥ 1.74-fold) between the chemosensitive (OE21 & OE33) and chemoresistant (KYSE450 & OE19) oesophageal cancer cell lines. **b** Real-Time PCR was used to assess the expression levels of miR-193b in these cell lines. RNU6B was used for normalisation and expression levels were quantified using 2^-ΔΔCp^

Supervised clustering analysis (*p* < 0.005) identified six miRNAs that were more highly expressed in the apoptotic chemosensitive cells and two miRNAs that had higher expression in the autophagy chemoresistant cells (≥ 1.74-fold change) (listed in Fig. 1a). Of all the miRNAs identified in this screen, miR-193b had the greatest level of differential expression between the two groups and thus was investigated further. Real-Time PCR confirmed that miR-193b was approximately six fold more highly expressed in the chemosensitive (OE21 & OE33) cell lines compared to the chemoresistant cell lines (KYSE450 & OE19) (Fig. 1b).

### Overexpression of miR-193b in KYSE450 cells increases their sensitivity to 5-FU

**Fig. 2:**
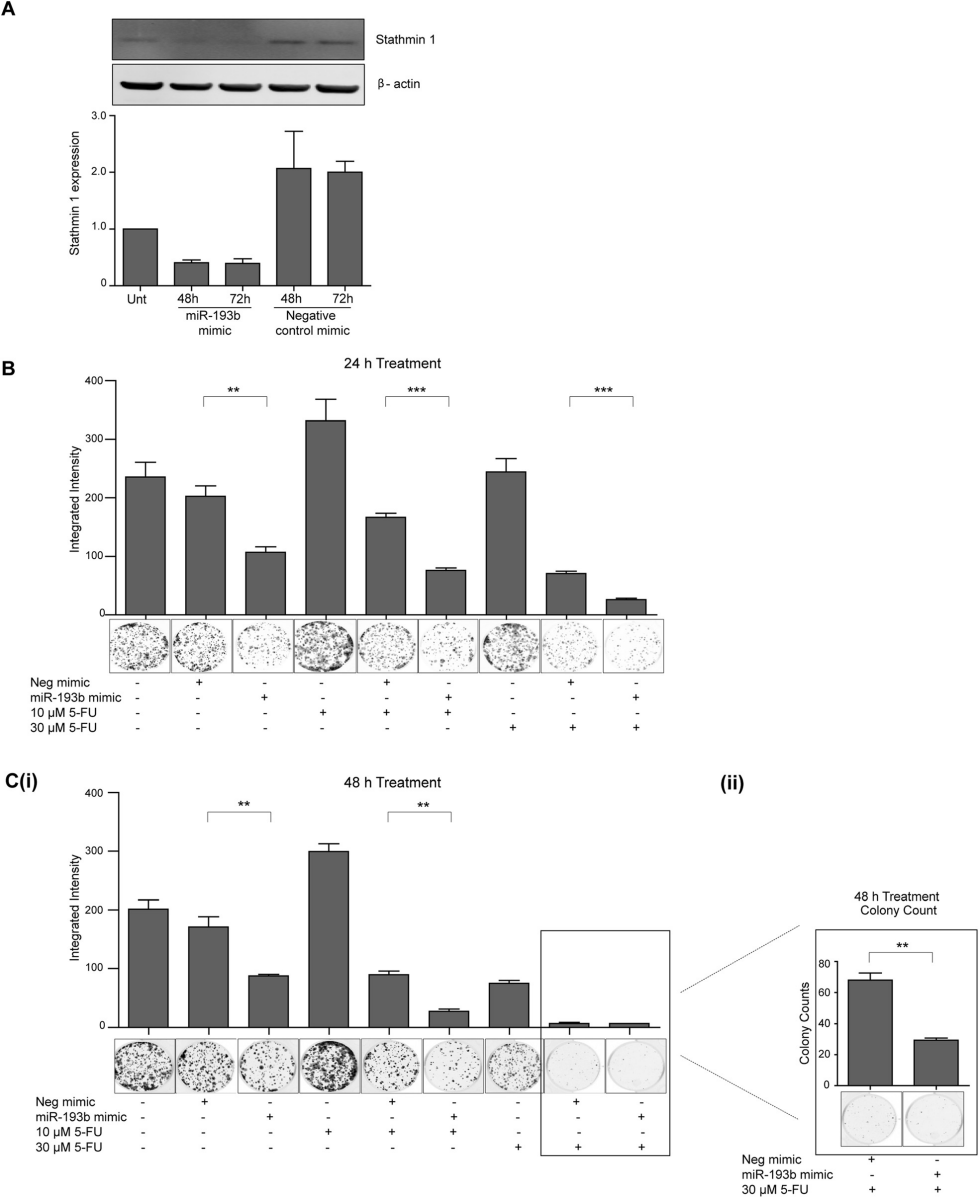
Examination of the consequences of miR-193b overexpression on recovery of KYSE450 oesophageal cancer cells. **a** KYSE450 cells were transfected with miR-193b mimic or negative control mimic (5 nM) and were assessed for expression of stathmin 1 (miR-193b target) by western blotting at 48 and 72 h post-transfection. β-actin was used as a loading control. Stathmin 1 levels were normalised to actin and quantification is shown in the lower panel. Data is presented as mean +/- S.E.M (*n* = 2). KYSE450 cells were transfected with miR-193b mimic or negative control (5 nM) and 24 h later were treated with 5-FU (10 μM or 30 μM) for 24 (**b**) and 48 (**c**) hours. Drug-treated and control cells were counted and 1500 KYSE450 cells were re-seeded in triplicate wells without drug and allowed to grow for 12 days. Colonies were fixed and counted using the Odyssey Infrared Imaging System (Li-Cor). **(C ii)** The number of colonies observed for negative control mimic + 30 μM 5-FU and miR-193b mimic + 30 μM 5-FU treatments (*boxed*) were below the detection limit of the Odyssey Imaging system and were manually counted. Representative wells are shown. Asterisks indicate statistical significance determined by *t*-test (**p < 0.01, ***p < 0.001)

As miR-193b is more highly expressed in the apoptotic/chemosensitive cell lines, we evaluated the functional consequences of miR-193b overexpression (using mimic technology) in the chemoresistant autophagy inducing KYSE450 cells. KYSE450 cells were transfected with a miR-193b mimic or negative control mimic (5 nM) and treated 24 h later with 5-FU (10 μM or 30 μM) for up to 48 h. Equal numbers of viable cells from each treatment group were then re-seeded and incubated for up to 12 days in the absence of drug to assess recovery. Overexpression of miR-193b was confirmed by examining the expression levels of stathmin 1. Stathmin 1 is a previously validated target of miR-193b (i.e. increased expression of miR-193b decreases stathmin 1 expression) [23]. Protein levels of stathmin 1 were reduced in miR-193b mimic transfected cells compared to negative control cells for up to 72 h post-transfection (Fig. 2a).

Overexpression of miR-193b mimic alone reduces the colony re-growth of KYSE450 cells compared to expression of the negative control mimic (**p = 0.0091). When miR-193b overexpressing cells were treated with 5-FU for 24 h, significantly fewer colonies re-grew when compared to 5-FU treated cells expressing the negative control mimic (***p = 0.0004 and ***p = 0.0007 for 10 and 30 μM 5-FU respectively) (Fig. 2b). A similar pattern was observed following 48 h treatment (Fig. 2c). Fewer colonies were observed following miR-193b overexpression when treated with 30 μM 5-FU for 48 h, but these differences were below the detection levels of the instrument, therefore these colonies were manually counted and these data are presented as Fig. 2c (ii). These results suggest that miR-193b expression can have a significant impact on viability and chemosensitivity.

### MiR-193b may mediate its effect on chemosensitivity through negative regulation of Stathmin 1

Several of the freely available miRNA target prediction software programs were used to identify potential targets of miR-193b. These included Targetscan, Pictar, miRDb, Targetminer, miRSearch 3.0, Diana-microT, MiRwalk and MiRTarBase. These programs predicted several hundred downstream effectors of miR-193b, with a limited number of genes in common (data not shown). Stathmin 1 was identified by five prediction software programs and has been previously reported to be negatively regulated by miR-193b [23, 24]. Functionally, it has been associated with the destabilisation and disassembly of microtubules [17] and silencing stathmin 1 (using adenovirus technology) in combination with 5-FU was reported to increase apoptosis and decrease clonogenic survival of prostate cancer cells [25].

**Fig. 3:**
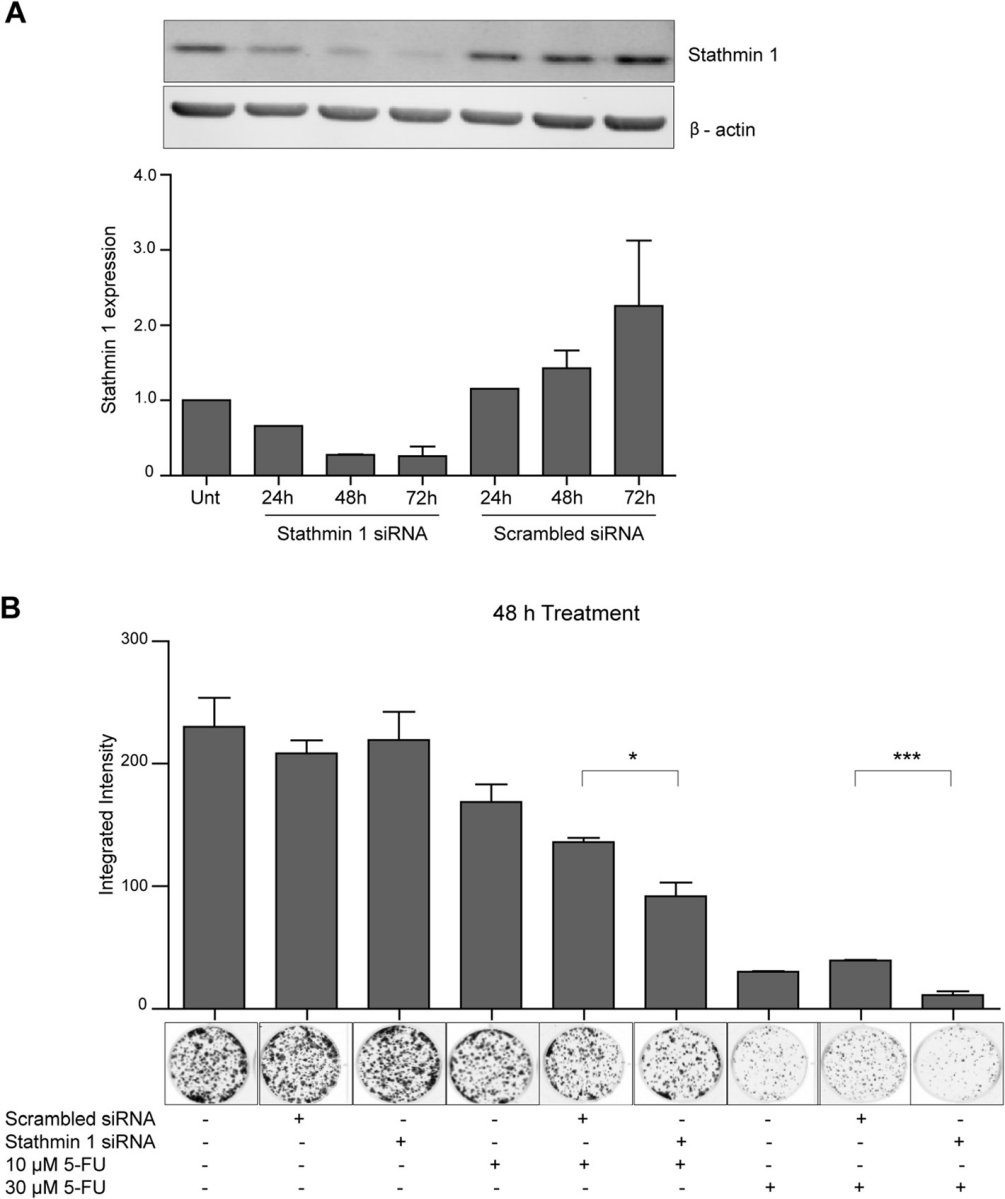
Evaluation of the consequences of Stathmin 1 knockdown on recovery of KYSE450 oesophageal cancer cells. **a** KYSE450 cells were transfected with stathmin 1 siRNA or scrambled siRNA (20 nM) and stathmin 1 levels were assessed by western blotting at 24, 48 and 72 h post-transfection. β-actin was used as a loading control. Stathmin 1 levels were normalised to actin and quantification is shown in the lower panel. Data is presented as mean +/- S.E.M (*n* = 2), (the 24 h time point is not a duplicate. However, 48 and 72 h transfection time points correspond to 24 and 48 h time points in the clonogenic assays below). **b** KYSE450 cells were transfected with stathmin 1 siRNA or scrambled siRNA (20 nM) and 24 h later were treated with 5-FU (10 μM or 30 μM) for 48 h. Drug-treated and control cells were counted and 1500 KYSE450 cells were re-seeded in triplicate wells without drug and allowed to grow for 12 days. Colonies were fixed and counted using the Odyssey Infrared Imaging System. Representative wells are shown. Asterisks indicate statistical significance determined by *t*-test (*p < 0.05, ***p < 0.001)

We therefore examined the effects of siRNA knockdown of stathmin 1 in KYSE450 cells. Western blot analysis showed that stathmin 1 levels were reduced up to 72 h following transfection compared to the corresponding scrambled control (Fig. 3a). Reduced clonogenic survival was observed in KYSE450 cells transfected with stathmin 1 siRNA and treated with 5-FU for 48 h compared to the equivalent scrambled siRNA controls treated with 5-FU (*p = 0.0202, ***p = 0.0009 for 10 and 30 μM 5-FU respectively) (Fig. 3b). A significant difference was not observed at 24 h (data not shown). Western blot analysis indicated greater silencing of stathmin 1 at 48 h which may account for this (Fig. 3a). These results suggest that knockdown of stathmin 1 can promote sensitivity of KYSE450 cells to 5-FU. Thus, miR-193b may mediate some of its effects on chemosensitivity in KYSE450 cells through the negative regulation of the stathmin 1 transcript.

### MiR-193b overexpression and 5-FU treatment does not activate apoptosis

**Fig. 4:**
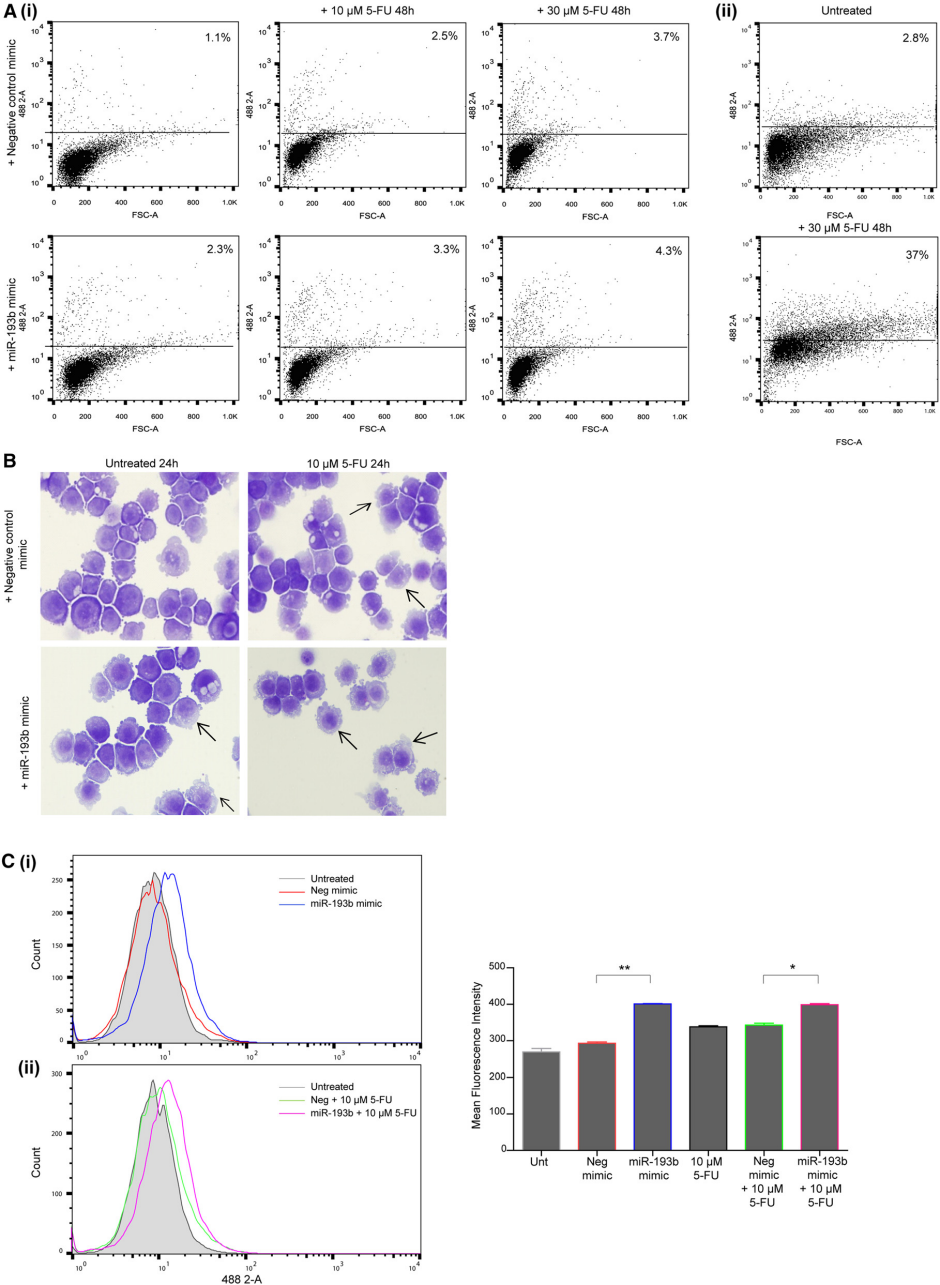
Examination of the effects of miR-193b overexpression on active caspase 3, cell morphology and Cyto-ID. **a** Active caspase 3 levels were assessed by FACS analysis. **(i)** KYSE450 cells were transfected with negative control mimic or miR-193b mimic (5 nM) and the following day were treated with 5-FU (10 μM or 30 μM) for 48 h. An upward shift in the levels of Alexa-Fluor 488 represented an increase in the number of cells with activated caspase 3 (percentage of cells with activated caspase 3 is shown). Upper panels – negative control transfected cells treated with 10 μM (*middle*) or 30 μM 5-FU (*right panel*). Lower panels – miR-193b transfected cells treated with 10 μM (*middle*) or 30 μM 5-FU (*right panel*). **(ii)** OE21 cells were treated with 30 μM 5-FU for 48 h (lower panel). **b** Morphological features of negative control mimic or miR-193b (5 nM) transfected KYSE450 cells. Twenty four hours following transfection, cells were treated with 5-FU (10 μM) for 24 h and compared to untreated transfected controls. Autophagic cells are indicated with arrows (×40 magnification). **c** KYSE450 cells were transfected with negative control mimic or miR-193b mimic (5 nM). Twenty-four hours following transfection, cells were treated with 5-FU (10 μM) for 24 h and then assessed for autophagy induction using the Cyto-ID autophagy detection assay. Representative image of FACS analysis of Cyto-ID levels of **(i)** untreated, negative control mimic and miR-193b transfected cells and **(ii)** untreated, negative control mimic and miR-193b mimic cells treated with 10 μM 5-FU. Right hand panel - mean fluorescence intensity of all treatments. Asterisks indicate statistical significance determined by *t*-test (*p < 0.05, **p < 0.01)

We further investigated the mechanism by which overexpression of miR-193b reduces cell viability. We assessed the levels of both apoptosis and autophagy in transfected cells. Flow cytometric analysis showed that overexpression of miR-193b did not increase the levels of active caspase 3 compared to the negative control mimic transfected cells. There was also no difference following 5-FU treatment for 24 (data not shown) or 48 h (Fig. 4a (i)). This is in contrast to OE21 cells which showed high levels of active caspase 3 activity following treatment with 30 μM 5-FU for 48 h (Fig. 4a (ii)). These data suggest that overexpression of miR-193b in KYSE450 cells does not result in apoptosis induction and is therefore unlikely to be responsible for the loss of survival observed in combination with 5-FU treatment.

MiR-193b overexpressing KYSE450 cells treated with 5-FU (10 μM) for 24 h showed a greater accumulation of vesicles in the cytoplasm when compared to negative control mimic treated cells (Fig. 4b – lower right panel, indicated by arrows). Apoptosis was rarely observed, in keeping with the caspase 3 data. Similar results were seen with 30 μM 5-FU treatment (data not shown).

The vesicular morphology observed suggested that autophagy may be induced following miR-193b overexpression or with 5-FU treatment. Autophagy vesicles were quantified using the Cyto-ID reagent, which specifically fluoresces in autophagic vesicles. Untransfected (shaded histogram) and negative control transfected cells (red overlay) had equivalent levels of fluorescence (Fig. 4c (i); and graphed to the right). KYSE450 cells ectopically expressing miR-193b for 48 h (Fig. 4c (i); blue overlay) showed increased levels of Cyto-ID fluorescence (**p = 0.0018). Autophagy was also assessed in cells treated with 5-FU (10 μM) for 24 h. Cells transfected with the miR-193b mimic and 5-FU showed significantly higher levels of autophagy compared to the negative control mimic transfected cells treated with 5-FU (*p = 0.0102) (Fig. 4c (ii); pink overlay compared to green). Similar results were seen with 30 μM 5-FU (data not shown).

### MiR-193b overexpression affects autophagy flux

**Fig. 5:**
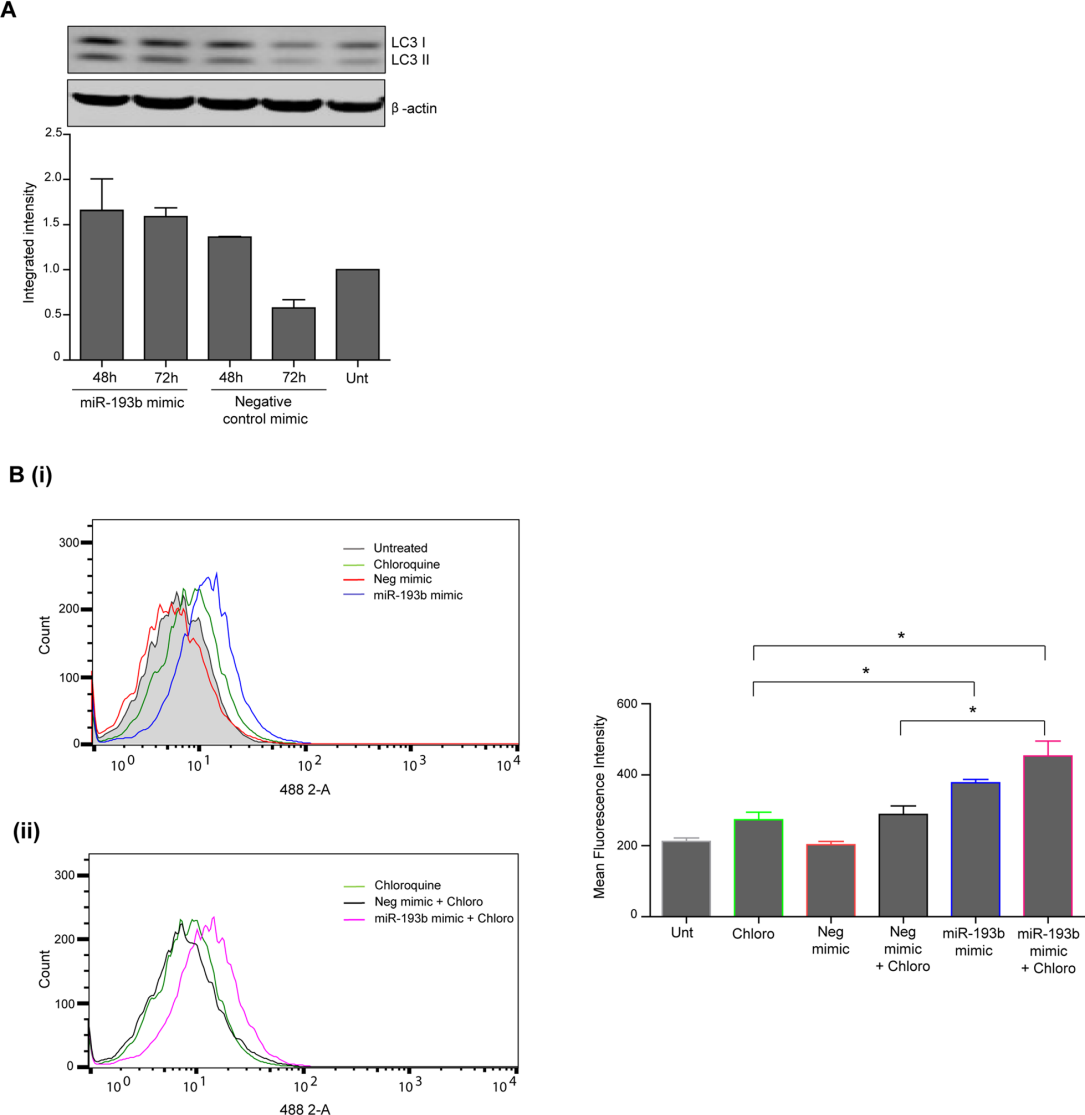
Evaluation of autophagy levels and flux analysis following miR-193b overexpression in KYSE450 oesophageal cancer cells. **a** Western blot analysis of LC3 II (autophagy marker) expression at 48 and 72 h post-transfection with miR-193b mimic or negative control mimic (5 nM). β-actin was used as a loading control. LC3 II levels were normalised to actin and quantification is shown in the lower panel. Data is presented as mean +/- S.E.M (*n* = 2). **b** KYSE450 cells were transfected with negative control mimic or miR-193b mimic (5 nM). Twenty-four hours following transfection, cells were treated with chloroquine (10 μM) and cells were assessed for autophagy induction 48 h after treatment using the Cyto-ID autophagy detection assay. **(i)** Representative FACS analysis of Cyto-ID levels of untreated, chloroquine treated, negative control mimic and miR-193b transfected cells. **(ii)** Representative FACS analysis of chloroquine, negative control mimic and miR-193b mimic transfected cells treated with chloroquine. The right hand panel shows the corresponding mean fluorescence intensity of all treatments. Asterisks indicate statistical significance determined by *t*-test (*p < 0.05)

Autophagy induction following miR-193b overexpression was further assessed by examining the levels of LC3 II (autophagy marker). Higher levels of LC3 II expression were observed in miR-193b overexpressing cells compared to their corresponding negative control as detected by western blotting (Fig. 5a). Together with the Cyto-ID analysis, these results suggested that miR-193b overexpression increased the levels of autophagic vesicles in these cells.

We then assessed whether overexpression of miR-193b affected “autophagic flux” in KYSE450 cells. This term is used to incorporate all stages of the autophagy process, including formation of the autophagosome, transport of autophagic substrates to the lysosome and fusion and degradation inside the lysosome [26]. To distinguish between autophagy induction and vesicle accumulation solely due to lack of vesicle processing, chloroquine, a lysomotrophic agent was used to block autophagosome-lysosome fusion. In the presence of chloroquine, the levels of autophagosomes will not be further increased unless more autophagy has been initiated [27]. KYSE450 cells were transfected with the miR-193b mimic or negative control mimic and 24 h later were treated with chloroquine (10 μM) for 24 (data not shown) and 48 h (Fig. 5b (i) & (ii)). Cyto-ID was used to assess autophagy levels. As previously shown, untreated cells (shaded histogram) had equivalent levels of fluorescence as cells transfected with the negative control mimic (red overlay). Treatment with chloroquine alone (green overlay) caused an increase in the levels of vesicles, consistent with an obstruction of vesicle turnover. A further shift in fluorescence was observed with miR-193b overexpression (blue overlay) (*p = 0.012) (Fig. 5b (i)). Graphical representation of the mean fluorescent intensities is shown on the right hand panel.

Chloroquine was then combined with mimics. Levels of autophagosomes were equivalent in the negative control mimic transfected cells plus chloroquine (black overlay) to the levels observed in cells treated with chloroquine alone (green overlay) (Fig. 5b (ii)) and graphed to the right). Importantly, the addition of chloroquine to the miR-193b transfected cells induced a further accumulation of autophagosomes (pink overlay), beyond that observed with either chloroquine alone (green overlay) (*p = 0.0195) or the combination of negative mimic and chloroquine (black overlay) (*p = 0.0277) (Fig. 5b (ii). Similar results were also observed at 24 h (data not shown). These results indicate that overexpression of miR-193b enhances autophagy in the KYSE450 cells.

### MiR-193b enhances chemosensitivity by inducing non-apoptotic cell death

**Fig. 6:**
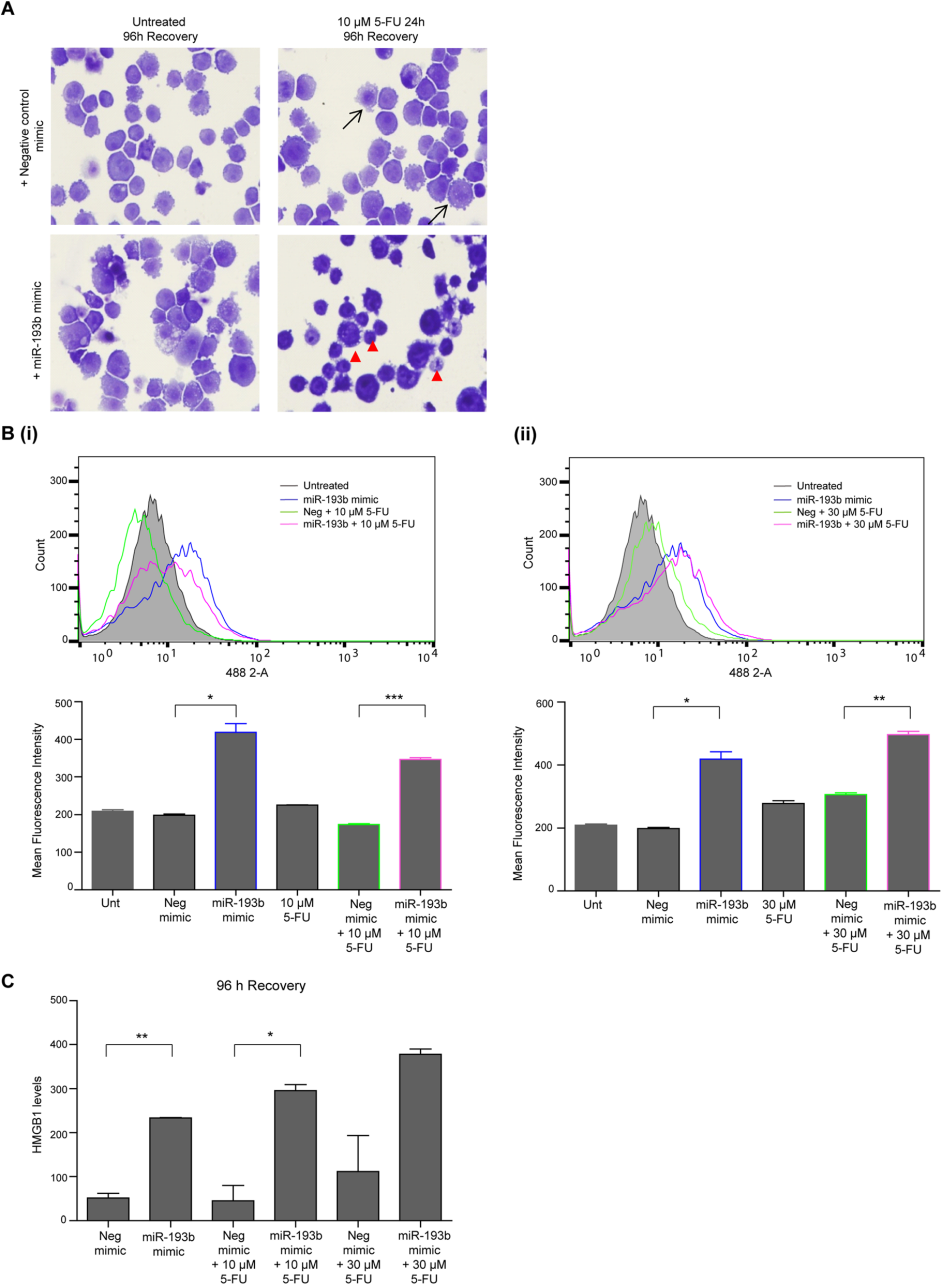
Analysis of morphology, autophagy levels and HMGB1 release from recovering 5-FU treated miR-193b overexpressing cells. KYSE450 cells were transfected with negative control mimic or miR-193b (5 nM). Twenty four hours following transfection, cells were treated with 5-FU (10 μM) for 24 h, following which these cells were allowed to recover in drug free media for 96 h. **a** Morphological features of negative control mimic or miR-193b transfected KYSE450 cells which had been treated with 5-FU for 24 h and left to recover 96 h. Autophagy and non-apoptotic/type II cell death are shown by arrows and arrowheads respectively (×40 magnification). **b** Cyto-ID analysis of the KYSE450 cells at the 96 h recovery time-point. Upper panel shows FACS analysis of untreated, miR-193b mimic transfected cells in the presence or absence of 5-FU (10 **(i)** or 30 μM **(ii)**) and negative control mimic 5-FU treated cells (10 μM **(i)** or 30 **(ii)**). Negative control mimic (fluorescence levels equivalent to untreated) and 5-FU alone treated cells (fluorescence levels equivalent to negative control mimic treated with 5-FU) were omitted from these images to aid visualisation of the other treatments. The lower panels show corresponding mean fluorescence intensity of all treatments. **(C)** At the 96 h recovery time-point, media was removed from all treated cells to assess HMGB1 levels. HMGB1 levels were quantified using a HMGB1 ELISA. Asterisks indicate statistical significance determined by *t*-test (*p < 0.05, **p < 0.01, ***p < 0.001)

The significant difference observed in colony formation assays between miR-193b mimic transfected cells treated with 5-FU and the corresponding negative control cells (Fig. 2b & c) could not be explained by the induction of apoptosis at the 24 h treatment time-point (Fig. 4). Therefore, we examined the response of cells treated for 24 h followed by an extended incubation (96 h) in drug free media (analogous to clonogenic assays). There were significantly more floating cells in the 5-FU treated miR-193b transfected cells compared to the equivalent negative control treated cells and also clear differences in cell morphology. The predominant morphology of 5-FU treated miR-193b mimic transfected KYSE450 cells was non-apoptotic cell death (Fig. 6a – lower right hand panel, indicated by arrowheads). In contrast, the 5-FU treated negative control mimic KYSE450 cells were healthy with some vesicles (Fig. 6a – upper right hand panel). Similar results were seen when miR-193b was overexpressed and treated with 5-FU for 48 h and left to recover for 96 h (data not shown). These results suggest that miR-193b overexpression promotes a delayed non-apoptotic cell death in response to 5-FU treatment and this may account for its impact on clonogenic survival.

Quantitation of autophagy vesicles by Cyto-ID analysis was also assessed in KYSE450 cells transfected with the miR-193b mimic, then treated with 5-FU for 24 h and left to recover in drug free media for 96 h. As previously shown (Fig. 4c), there was a forward shift in fluorescence for KYSE450 cells transfected with the miR-193b compared to the negative control cells. This increase in fluorescence for miR-193b transfected cells (blue bar) compared to negative control mimic cells was still evident at the 96 h recovery time point (*p = 0.0115) (Fig. 6b (i) & (ii)). This 96 h recovery data shows that when combined with 5-FU, there were significantly higher levels of autophagy in the miR-193b transfected cells compared to negative control mimic cells (***p = 0.0008 and **p = 0.0039 for 10 and 30 μM 5-FU respectively) (Fig. 6b (i) & (ii)). This data suggested that co-incident with loss of viability, miR-193b transfected cells have higher levels of autophagic vesicles. This may be associated with a higher stress level in the cells (and is unrelated to the death mechanism) or it is possible that it is associated with the non-apoptotic cell death observed at this time point.

Collectively, these results suggested that 5-FU treatment of KYSE450 cells overexpressing miR-193b resulted in higher levels of non-apoptotic cell death. To quantify this cell death, we assessed the levels of HMGB1 release. HMGB1 release is a well-known marker of various forms of cell death including apoptosis, autophagic cell death and necrosis in response to chemotherapy (reviewed in [28]). We assayed levels of HMGB1 release from KYSE450 cells ectopically expressing miR-193b or the negative control mimic, treated with 5-FU for 24 h and left to recover in drug free media for 96 h. At the 96 h recovery time-point, HMGB1 levels were at least three times higher in miR-193b overexpressing cells or miR-193b overexpressing cells which had been treated with 5-FU compared to the equivalent negative control cells (Fig. 6c). Therefore, overexpression of miR-193b caused a delayed release of HMGB1, which is normally associated with cell death.

## Discussion

In this study, we have shown that expression of miR-193b increases the chemosensitivity of oesophageal cancer cells. Our results suggest that miR-193b may affect chemosensitivity, in part, by decreasing the expression of stathmin 1. Overexpression of miR-193b results in the induction of autophagy. Heightened levels of autophagy may be important for some of the functional activities of miR-193b or it may be an indirect consequence of its activity.

To date, several miRNAs including miR-141, miR-200c, miR-148a, miR-296, miR-27a, miR-483 and miR-214 have been identified to play a role in oesophageal cancer development and or in determining the responsiveness of these cancer cells to chemotherapeutics [29–31]. Our data suggests that miR-193b can now be added to this list of miRNAs whose expression affects the sensitivity of oesophageal cancer cells to chemotherapy.

MiR-193b has been postulated to be a tumour suppressor in several cancers including gastric cancer due to its lower expression in malignant tissue compared to the corresponding normal tissue [24, 32–35]. Putative functions identified to date for miR-193b include the negative regulation of proliferation, cell cycle control, migration and invasion of hepatocellular carcinoma cells and non-small cell lung cancer cells [34, 36]. MiR-193b has previously been linked to enhancing chemosensitivity. In melanoma cells, overexpression of miR-193b reduced Mcl-1 expression and sensitised these cells to ABT-737 (BH3 mimetic) induced apoptosis [33]. In hepatitis B virus associated hepatocellular carcinoma cells, enhancing miR-193b expression lowered the IC50 of these cells to sorafenib treatment and apoptosis was triggered in these cells [37]. Our research is in agreement with these studies showing the importance of miR-193b for chemosensitivity. Recently, a study of two oesophageal cancer cell lines (OE19 and KYSE410) identified several miRNAs associated with chemoresistance. In that study, they found that OE19 cells which were engineered to be resistant to 5-FU had higher expression levels of miR-193b compared to their parental cells [38]. This is in contradiction to our research which showed that chemosensitive cells had higher expression levels of miR-193b. The mechanism of chemoresistance was not investigated in that analysis. However, our work is the first reported study showing that miR-193b overexpression in combination with 5-FU treatment results in the induction of autophagy and non-apoptotic cell death. MiR-193b’s functional role has never been previously investigated in oesophageal cancer cells and our work indicated that overexpression of this miRNA in combination with 5-FU treatment is effective in sensitising these drug resistant cells.

Several reports have shown that miR-193b directly targets Mcl-1 [33, 37]. The authors report that suppressing the anti-apoptotic protein Mcl-1 induced apoptosis in these cells. However, in our study, overexpression of miR-193b did not trigger apoptosis in our cells and consequently alternative targets of miR-193b were investigated. We identified that miR-193b may influence chemosensitivity through the negative regulation of stathmin 1. Silencing of stathmin 1 could at least partially re-capitulate the enhanced sensitivity to 5-FU that was observed with the overexpression of miR-193b. MiR-193b has been shown to regulate the expression of stathmin 1 and consequently affected tumour growth and metastasis in pancreatic cells [24]. Stathmin 1 has also been reported to affect cell proliferation and migration capabilities of melanoma cells [23]. Knockdown or low levels of stathmin 1 could sensitise breast cancer cells to paclitaxel, vinblastine or docetaxel respectively demonstrating its importance in determining the responsiveness of cancer cells to chemotherapeutics [16, 18]. High expression levels of stathmin 1 have been associated with lymph node metastasis and increased malignancy in oesophageal adeno and squamous cell carcinoma [39, 40]. Of note, stathmin 1 has also been putatively identified as an autophagy regulator, potentially through its importance for microtubule dynamics [12, 41]. As miR-193b potentially influences the expression of greater than 200 genes, it is likely that there are additional targets of this miRNA which may, at least in part, affect chemosensitivity.

There have been no previously reported studies linking miR-193b and autophagy. In our study, we showed that overexpression of miR-193b results in an increase of LC3 II and autophagic vesicles (as determined by Cyto-ID) and also affected autophagy flux. It is unclear from this study whether increased expression of miR-193b directly or indirectly affected autophagy. To date, there have been far in excess of twenty miRNAs implicated directly in the regulation of autophagy and this list is increasing rapidly [11–14]. The impact of de-regulation of these miRNAs on autophagy and on cellular processes including cell death is only beginning to emerge. Further analysis could add miR-193b to this list. For example, miR-23b has been shown to directly target ATG12b. Overexpression of this miRNA sensitised pancreatic cells to radiotherapy by inhibiting radiation induced autophagy. The mechanism which resulted in the reduced survivability of these cells was not delineated [42]. In melanoma cells, miR-290-295 cluster suppressed autophagic cell death in response to glucose starvation through the down-regulation of ATG7 and ULK1 [43].

In our study, it is possible that miR-193b’s relationship with stathmin 1 (potential autophagy regulator) may be important for the autophagy induction observed following miR-193b overexpression. However, it remains a possibility that miR-193b increases the autophagy level in these cells through an indirect mechanism. For example, we could postulate that enhanced miR-193b expression could elevate accumulation of macro-molecular structures or organelles (e.g. through effects on microtubules) and consequently induces autophagy (indirect mechanism). The exact relationship between miR-193b and autophagy remains to be fully elucidated.

## Conclusions

MiR-193b is a regulator of chemosensitivity, potentially through the regulation of autophagy. The enhanced cell death associated with miR-193b overexpression suggests that this novel miRNA has a potential application in oesophageal cancer therapy.

## Abbreviations

5-FU: 5-Fluorouracil
HMGB1: high mobility group box 1
miRNA: microRNA
siRNA: small interfering RNA
